# Folic Acid Adjustive Polydopamine Organic Nanoparticles Based Fluorescent Probe for the Selective Detection of Mercury Ions

**DOI:** 10.3390/polym15081892

**Published:** 2023-04-14

**Authors:** Lijuan Chen, Changchang Chen, Yehan Yan, Linlin Yang, Renyong Liu, Jiajia Zhang, Xin Zhang, Chenggen Xie

**Affiliations:** 1 College of Materials and Chemical Engineering, West Anhui University, Lu’an 237012, China; lijuanch88@wxc.edu.cn (L.C.);; 2 School of Energy Materials and Chemical Engineering, Hefei University, Hefei 230601, China; 3 School of Life Sciences, Anhui Agricultural University, Hefei 230036, China

**Keywords:** polydopamine, folic acid, mercury ions, fluorescence probe

## Abstract

Polydopamine fluorescent organic nanomaterials present unique physicochemical and biological properties, which have great potential application in bio-imaging and chemical sensors. Here, folic acid (FA) adjustive polydopamine (PDA) fluorescent organic nanoparticles (FA-PDA FONs) were prepared by a facile one-pot self-polymerization strategy using dopamine (DA) and FA as precursors under mild conditions. The as-prepared FA-PDA FONs had an average size of 1.9 ± 0.3 nm in diameter with great aqueous dispersibility, and the FA-PDA FONs solution exhibit intense blue fluorescence under 365 nm UV lamp, and the quantum yield is ~8.27%. The FA-PDA FONs could be stable in a relatively wide pH range and high ionic strength salt solution, and the fluorescence intensities are constant. More importantly, here we developed a method for rapidly selective and sensitive detection of mercury ions (Hg^2+^) within 10 s using FA-PDA FONs based probe, the fluorescence intensities of FA-PDA FONs presented a great linear relationship to Hg^2+^ concentration, the linear range and limit of detection (LOD) were 0–18 µM and 0.18 µM, respectively. Furthermore, the feasibility of the developed Hg^2+^ sensor was verified by determination of Hg^2+^ in mineral water and tap water samples with satisfactory results.

## 1. Introduction

Fluorescent organic nanoparticles (FONs) have attracted extensive interest in many fields, owing to their excellent optical property, diffusion capability, biocompatibility and photostability, which gives them potential for application in fields such as sensing and bioimaging. [1,2,3,4,5]. Polydopamine (PDA) based organic nanomaterials have received lots of attention due to their excellent biocompatibility, strong adhesive properties, being easy to prepare, metal ion chelating, and chemical reactivity, among other properties [6,7,8,9,10]. The general synthesis of PDA nanoparticles includes the dopamine oxidative self-polymerization into quinone and 5,6-dihydroxyindole under alkaline conditions at room temperature, resulting in a chemical disorder structure with covalent polymerization and π-π stacking interaction between planar aromatic rings in PDA [11,12,13], and then leads to the aggregation caused fluorescence quenching (ACQ) [14]. Actually, PDA nanoparticles exhibits weak and wavelength-dependent fluorescence under UV irradiation, the dopamine oligomers are the main source of fluorescence, and the high degree of polymerization of dopamine is unfavorable [15,16]. Therefore, it is intriguing to prepare the polydopamine fluorescent organic nanoparticles (PDA FONs) by suppressing the degree of polymerization or reducing the π-π stacking interaction in PDA [16,17]. The first PDA FONs were reported in 2012, where hydrogen peroxide (H_2_O_2_) was used to oxidize PDA nanoparticles and regulate the π-π stacking interaction, which could enhance the fluorescence intensity of PDA nanoparticles [18]. Whereafter, other oxidizing agents such as: KMnO_4_, MnO_2_, CoOOH nanosheets, and ammonium persulfate were used to prepare PDA FONs [19,20,21,22]. Beyond that, thiol- or amine-containing organic species, such as polyethyleneimine [23,24,25], glutathione [26], and glycine [17] were added in the DA self-polymerization process to prepare PDA FONs via Michael addition or Schiff base reaction to prevent dopamine cyclization. However, there are still some drawbacks in the preparation of PDA FONs, such as low quantum yields (less than 1.2%), complex synthesis processes, and involvement of H_2_O_2_ (hazardous agents relatively). Therefore, to developing facile, effective, and environmental-friendly strategies for PDA FONs is still in demand.

FA is a water-soluble vitamin, which is made up of pterin (PT), p-aminobenzoic acid, and L-glutamic acid moieties, and the PT group in folic acid could self-assemble to form a tetramer through hydrogen bonding or π-π interactions [27,28,29]. Beyond that, the PT group also could act as a π acceptor due to the structure of planar N heterocycle, which can interact with π donors [30], for example, the π-π interactions between FA and N-(9-fluorenylmethyloxycarbonyl)-3,4-dihydroxy-l-phenylalanine are identified as the main driver to form the twisted fibers [31]. Jin et al. have demonstrated the dramatic effect of FA on the PDA nanostructure, and supposed that the π-π interactions and hydrogen bonding between FA and PDA may result in the PDA nanofibers formation [32]. Actually, several reaction parameters such as reaction time, temperature, the concentration and ratios of DA to FA may affect the PDA nanostructures; however, little attention has been paid in the investigation of the fluorescent property of PDA nanoparticles in this system.

**Scheme 1:**
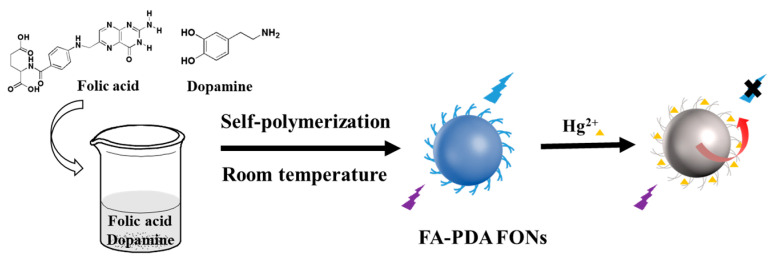
The synthesis and application of FA-PDA FONs.

Inspired by this, we used FA and DA as precursors to prepare the FA adjustive PDA fluorescent organic nanoparticles (FA-PDA FONs) by using a facile one-pot green method at room temperature (Scheme 1). High-resolution transmission electron microscopy (HRTEM) was used to characterize the morphology and nanoparticles size of the FA-PDA FONs. X-ray diffraction (XRD), X-ray photoelectron spectra (XPS), Fourier-transform infrared spectra (FTIR), and zeta potential measurement were used to analyze the chemical structure, composition, and surface electric charge of the FA-PDA FONs. The spectral characteristics of the FA-PDA FONs were characterized by using ultraviolet visible (UV-vis) absorption spectra and photoluminescence spectra. Furthermore, an FA-PDA FONs probe was used for sensitive and selective detection of Hg^2+^. Moreover, the feasibility of the FA-PDA FONs based probe was also evaluated in real water samples.

## 2. Materials and Methods

### 2.1. Chemicals

Dopamine·HCl was obtained from sigma-Aldrich (St. Louis, MO, USA), FA and tris (hydroxymethyl) aminomethane (Tris) were both purchased from Aladdin (Shanghai, China). The metal salts (including NaCl; KCl; BaCl_2_; MgCl_2_·6H_2_O; ZnCl_2_; CaCl_2_; Mn(CH_3_COO)_2_·4H_2_O; Al(NO_3_)_3_·9H_2_O; FeCl_3_·6H_2_O; NiCl_2_; Co(NO_3_)_2_; Cu(NO_3_)_2_·3H_2_O; Sr(NO_3_)_2_; GaCl_3_; Cd(NO_3_)_2_·4H_2_O; Pb(CH_3_COO)_2_·3H_2_O; Cr(NO_3_)_3_·9H_2_O; Hg(NO_3_)_2_·H_2_O; Na_2_HPO_4_·12H_2_O; Na_3_PO_4_·12H_2_O; NaNO_3_; NaNO_2_; NaSCN; NaHCO_3_; Na_2_S·9H_2_O; CH_3_COONa; Na_2_SO_4_; NaClO_3_; NaSH·xH_2_O; NaBr; Na_2_CO_3_; Na_2_SO_3_**)**, and the amino acids (including glycine (Gly), phenylalanine (Phe), leucine (Leu), alanine (Ala), serine (Ser), isoleucine (Ile), proline (Pro), threonine (Thr), glutamic acid (Glu), histidine (His), aspartic acid (Asp), lysine (Lys), arginine (Arg), cysteine (Cys), asparagine (Asn), and glutamine (Gln)) were obtained from Aladdin (Shanghai, China). Other chemicals used in the work were all obtained from Sinopharm Chemical Reagent (Shanghai, China). Pure water was homemade and used throughout the experiments.

### 2.2. Instruments

Transmission electron microscopy (TEM) was implemented with a JEM 2100 transmission electron microscope (JEOL, Kyoto, Japan) operating at an acceleration voltage of 200 kV. XPS spectra were recorded on a Kratos AXIS Supra+ XPS spectrometer (Kratos Analytical Ltd., Manchester, UK) for characterizing the chemical elemental composition. XRD pattern was carried out on a D8 Advanced diffractometer with Cu Kα radiation (Bruker, Billerica, MA, USA). UV-vis spectra were obtained on a UV-6100 spectrophotometer (Shanghai, China), and fluorescence spectra were recorded on a Shimadzu RF-5301PC fluorescence (Shimadzu, Kyoto, Japan). FTIR spectra were obtained by using the iS50 FTIR spectrometer (Thermo Fisher Scientific, Waltham, MA, USA). Zeta potential was measured by using a Dynamic Light Scattering instrument (Brookhaven Instruments Corporation, New York, USA).

### 2.3. Synthesis of FA-PDA FONs

FA-PDA FONs was prepared by self-polymerization of DA and FA at room temperature. Typically, 0.03 g folic acid was dissolved in 100 mL pure water at room temperature and stirred for 2 h, and then 0.015 g dopamine·HCl was added in the solution and stirred for 30 min, then Tris was used to adjust the pH of the reaction solution to 9, and stirred for 5 h in room temperature, after that, the resulting solution was aged for one week. Afterwards, the solution was filtered by 0.22 μm microfilter membrane, and dialyzed through porous cellulose bag (molecular weight cut off 1000 Da) for 4 h in pure water. Finally, the samples inside the dialysis bag were collected and dried by freeze-drying, and the complete details are given in Supplementary Information.

### 2.4. Quantum Yield

The quantum yield (QY) of FA-PDA FONs was calculated by using quinine sulfate (Φ_sr_ = 54%) as the standard reference material [33,34], and the complete details are given in Supplementary Information.

### 2.5. Fluorescence Selectivity of FA-PDA FONs for Detection of Hg^2+^

With the optimal experimental conditions, the response of FA-PDA FONs probe (10 μg/mL) to 18 metal ions (Co^2+^, Ni^2+^, Mn^2+^, Na^+^, K^+^, Ba^2+^, Sr^2+^, Ca^2+^, Mg^2+^, Zn^2+^, Cd^2+^, Cu^2+^, Cr^3+^, Al^3+^, Fe^3+^, Ga^3+^, Pb^2+^ and Hg^2+^), 15 anions (Cl^−^, Br^−^, NO_2_^−,^ NO_3_^−^, CH_3_COO^−^, CO_3_^2−^, SO_3_^2−^, SO_4_^2−^, SCN^−^, SH^−^, PO_4_^3−^, HCO_3_^−^, S^2−^, HPO_4_^2−^ and ClO_3_^−^), and 16 amino acids (Thr, Gly, Gln, Asn, Ile, Arg, Glu, Leu, Asp, Pro, Cys, Ala, Ser, Lys, His and Phe) were carried out, and the analyte concentration was 1 × 10^−3^ mol/L, respectively. For the experiment of selectivity, 10 μL FA-PDA FONs and 100 μL analyte were added to the sample solutions, and kept the total volume at 2000 μL. The fluorescence spectra of above systems were recorded with 360 nm excitation, and the excitation and emission slit width were both set at 10 nm. The fluorescence intensities of FA-PDA FONs with/without analyte were represented as I and I_0_, respectively.

### 2.6. Fluorescence Sensitivity of FA-PDA FONs toward Hg^2+^

For Hg^2+^ sensing, 10 μL FA-PDA FONs and 200 μL different concentrations of Hg^2+^ (0–100 μM) were added to the sample solutions, respectively, and the total volume kept at 2000 μL, then all the mixed samples were incubated at room temperature for 2 min. The changes in FA-PDA FONs fluorescence intensity were measured at 360 nm with increase in Hg^2+^ concentration, the Stern–Volmer plots with quenching efficiency as the *Y*-axis and Hg^2+^ concentration as the *X*-axis were analyzed, and the correlation could be expressed by Formula (1) [1,35]

(I_0_ − I)/I_0_ = k [M] (1)

Here, the calculated slope is expressed as k, and the concentration of Hg^2+^ is expressed as [M].

## 3. Results and Discussion

### 3.1. Synthesis of FA-PDA FONs

**Figure 1:**
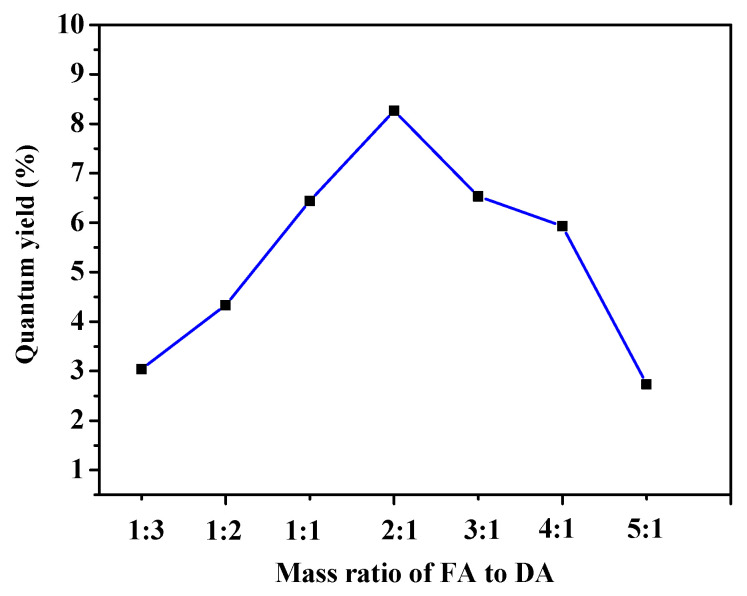
The QYs of the prepared FA-PDA FONs samples.

FA may be involved in the stacking of PDA by π-π interactions/hydrogen bonding, and some new aggregated nanostructures of PDA were generated (such as: nanobelts and nanofibers) in the dopamine/folic acid system [32]. The mass ratio of FA/DA, concentration, and reaction time of the system were the key factors in the outcome. In this work, the mass ratio of FA to DA were designed from 1:3 to 5:1, and the reaction time was shortened, and then we found that the reaction solutions emitted blue fluorescence under 365 nm UV lamp, and the QYs of the samples were measured, as shown in Figure 1 and Figure S1. Obviously, when DA is more than FA, the QY is lower, which means the aggregation of planar aromatic rings in PDA and leads to ACQ effect, and the QYs increase along with the increase in FA, it indicates that the enough FA could join in the DA self-polymerization, and affect the structure of PDA, when the mass ratio of DA to FA is 1:2, the QY of products is highest (8.27%). Hence, the FA-PDA FONs were prepared by using mass ratio of 1:2 for subsequent study.

### 3.2. Characterization of FA-PDA FONs

**Figure 2:**
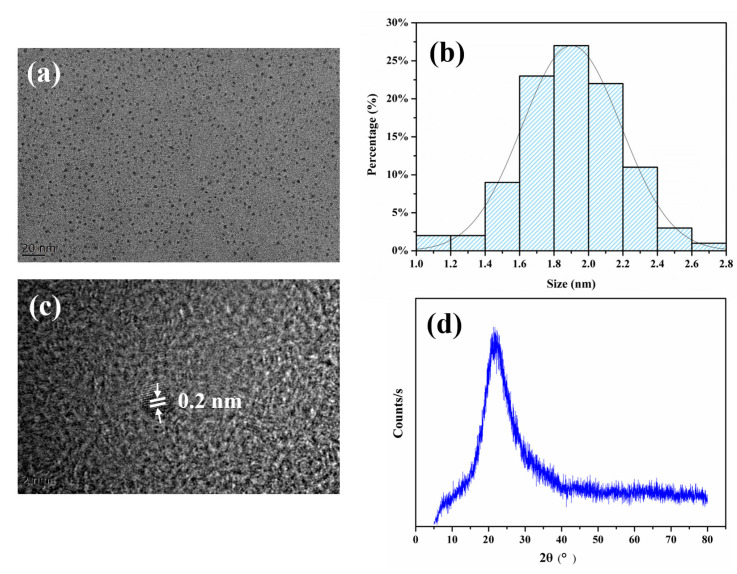
(**a**) TEM image of FA-PDA FONs. (**b**) Average size of FA-PDA FONs by intensity. (**c**) HRTEM image of FA-PDA FONs. (**d**) XRD pattern of FA-PDA FONs.

The morphology and particle size of FA-PDA FONs were characterized, as shown in Figure 2. Clearly, the prepared FA-PDA FONs are sphere-shaped (Figure 2a) and the size distribution are 1.0–2.8 nm with a uniform size of 1.9 ± 0.3 nm in diameter (Figure 2b), here, 100 particles were calculated for the statistics. The HRTEM image shows a crystal-like arrangement with an interlayer lattice fringe of 0.2 nm, which could be ascribed to the (100) in-plane lattice of graphene (Figure 2c) [9]. Actually, due to the disordered carbon network, FA-PDA FONs were amorphous, as evidenced by the absence of crystalline reflections with a broad feature spanning 10–30° 2θ (Figure 2d) [36].

**Figure 3:**
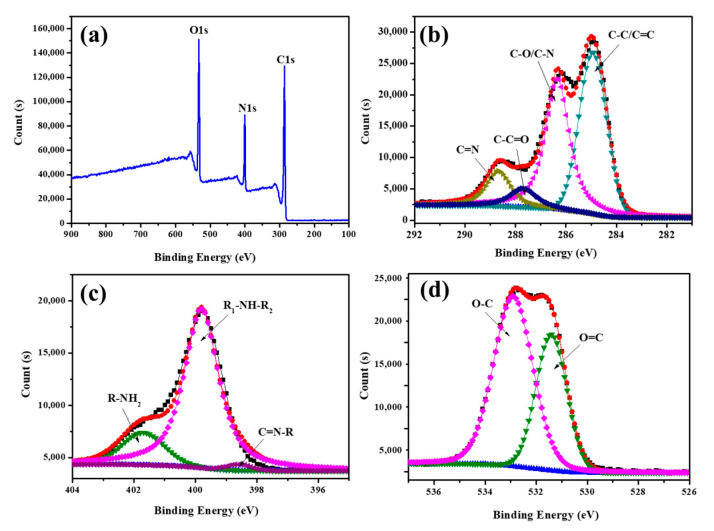
(**a**) XPS survey spectrum of FA-PDA FONs and high-resolution XPS spectra of (**b**) C1s peaks, (**c**) N1s peaks, and (**d**) O1s peaks, respectively.

The elemental composition and chemical bonds of the FA-PDA FONs were analyzed by XPS. In the full XPS spectrum of FA-PDA FONs, three prominent bands appeared at 285.1, 400.2, and 532.2 eV, corresponding to the C1s, N1s, and O1s (Figure 3a), and the relative content of elements was approximately 66.3% C, 15.0% N, and 18.7% O, respectively. For all we know that the N/C value of FA is 0.43, and the N/C value ranges of PDA is often from 0.08 to 0.17, interestingly, the N/C value of FA-PDA FONs here is 0.226, therefore, FA-PDA FONs may be hybrid materials and composed of folic acid, polydopamine, even Tris moieties [37]. As represented in Figure 3b, the C1s region can be integrated into four major component bands: C-C/C=C (284.82 eV), C-N/C-O (286.23 eV), C-C=O (287.70 eV), and C=N (288.63 eV) [5,38], correspondingly. In addition, the N1s region has three bands: C=N-R (398.6 eV), R_1_-NH-R_2_ (399.06 eV), and R-NH_2_ (401.87 eV), respectively [32], and the O1s region is fitted with two prominent bands: O-C (532.9 eV) and O=C (531.85 eV) [12,32].

**Figure 4:**
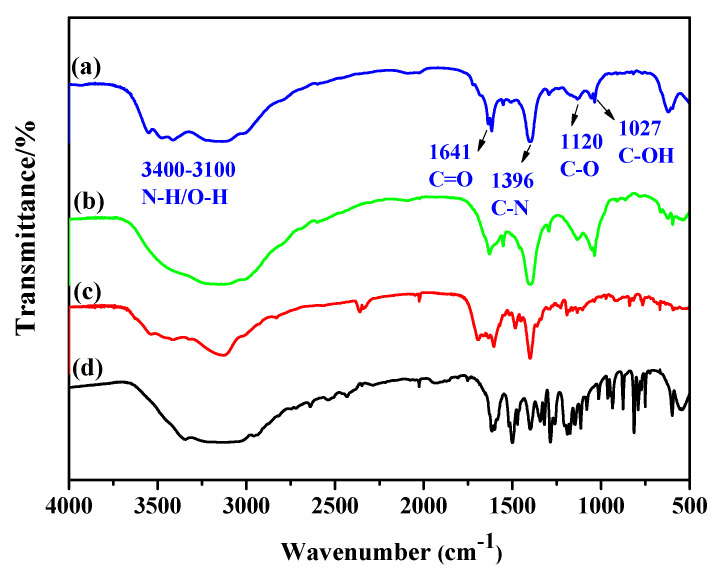
FTIR spectra of (**a**) FA-PDA FONs; (**b**) Polydopamine; (**c**) Folic acid; (**d**) Dopamine.

The FTIR spectra were collected to further understand the chemical structure of FA-PDA FONs, as shown in Figure 4. The broad peaks at 3400–3100 cm^−1^ are attributed to the stretching vibrations of O-H and N-H [39]. The peak at 1641 cm^−1^ is attributed to C=O stretching vibrations, and the peaks at 1396 and 1120 cm^−1^ are attributed to C-N stretching vibrations [10], and C-O bending vibrations [40], respectively. The peak at 1027 cm^−1^ is identified as the C-OH stretching vibration [41]. It is worth noting that the C-N intensities of FA-PDA FONs and polydopamine are obviously enhanced compared with dopamine, indicating they contain more repeated units of 5,6-dihydroxyindole after self-polymerization [16]. The above results were consistent with the XPS results, and also suggest that the FA-PDA FONs contain abundant hydrophilic functional groups, such as amino, hydroxyl, and carboxyl, which endows the FA-PDA FONs with great hydrophilicity and water dispersibility.

**Figure 5:**
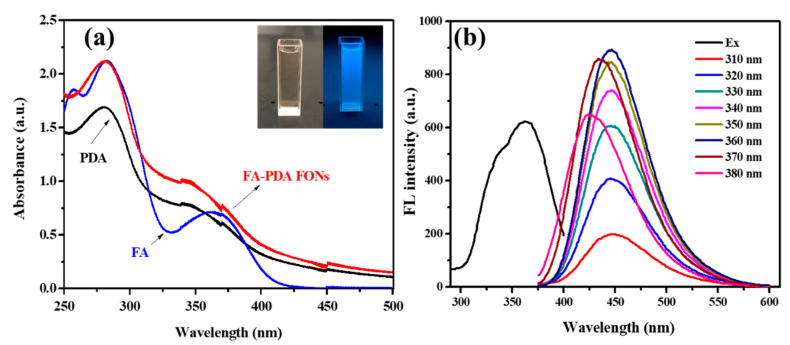
(**a**) UV-vis absorption spectra of FA, PDA and FA-PDA FONs. Inset shows the photographs of FA-PDA FONs probe under the natural light (left) and the irradiation of 365 nm UV lamp (right); (**b**) Excitation and fluorescence spectra of FA-PDA FONs with the excitation wavelength increased from 310 to 380 nm.

Next, the optical properties of as-prepared FA-PDA FONs were characterized. From the UV-vis absorption spectra showed in Figure 5a, we can see that two characteristic peaks appear at about 282 and 373 nm, corresponding to the π-π* electronic transitions of the C=C, and n-π* electronic transitions of C=O/C=N, respectively [42,43].

Insets show the pictures of aqueous solution of FA-PDA FONs under the irradiation of room light and 365 nm UV lamp. Obviously, under the irradiation of room light, the aqueous solution is very clear and limpid (left cell), indicating that the FA-PDA FONs possess great water dispersibility, when the aqueous solution is irradiated with 365 nm UV lamp, the strongly emitted blue fluorescence is observed (right cell), directly confirming the photoluminescence property of FA-PDA FONs solution. As shown in Figure 5b, when the excited wavelengths changed from 310 nm to 380 nm, the emission peak has a slight blue shift gradually, and the fluorescence (FL) intensity reached the maximum at 360 nm, and the phenomenon was reflected the excitation-dependent emission manner [44].

**Figure 6:**
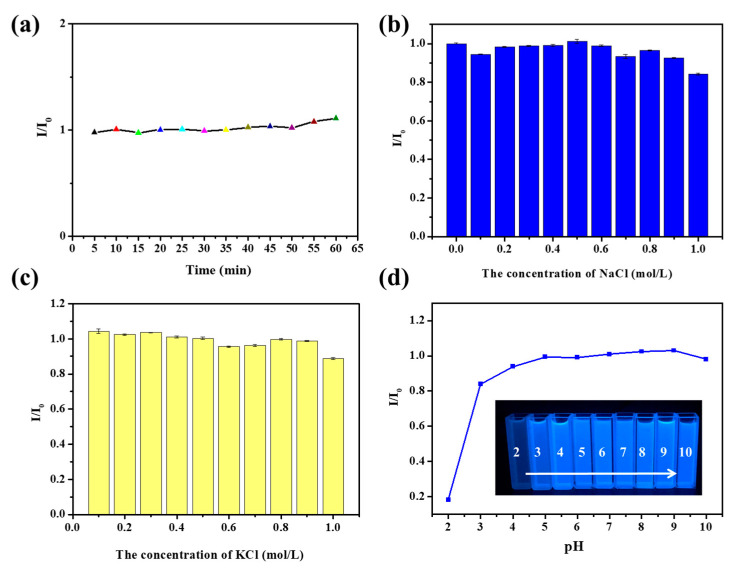
(**a**) FL intensities of FA-PDA FONs under 365 nm UV lamp with different illumination time; (**b**) FL intensities of FA-PDA FONs in different concentrations of NaCl; (**c**) FL intensities of FA-PDA FONs in different concentrations of KCl; (**d**) FL intensities of FA-PDA FONs in the different pH solution, inset shows the photographs of FA-PDA FONs in different pH solution under the irradiation of 365 nm UV lamp.

Furthermore, the FL intensity stability of FA-PDA FONs was investigated, as shown in Figure 6a, the FL intensity was unaffected by UV lamp consecutive irradiation at 365 nm for 60 min, indicating FA-PDA FONs have great ability against photobleaching. Figure 6b,c present the interference of salt concentration on the fluorescence intensity of FA-PDA FONs. It is obvious that even if the NaCl/KCl concentration is high to 1.0 mol/L, the FL intensity is almost unchanged, demonstrating the practicality of applying FA-PDA FONs in physiological salt conditions.

In addition, the prepared FA-PDA FONs exhibit strong, relatively stable FL intensity at pH 4.0 to 10.0, whereas at lower pH value, for instance, the FL intensity significantly decreased at pH = 2.0 (Figure 6d). Zeta potential of FA-PDA FONs were tested in different pH solution as shown in Figure S2. At pH = 2.0, zeta potential of the FA-PDA FONs was ~0 mV, and then changed to negative values at high pH condition. This phenomenon maybe attribute to the protonation/deprotonation of the hydroxyl, carboxyl, and amino groups of FA-PDA FONs, and the results is consistent with the reported works [45,46,47]. Obviously, the neutralization of the FA-PDA FONs surface or less surface charge is harmful for system stability and fluorescence emission. Actually, due to the aggregation of FA-PDA FONs at pH 2–3, precipitation was occurred after 3 days of storage, as shown in Figure S2.

### 3.3. Fluorescence Selectivity of FA-PDA FONs for Detection of Hg^2+^

**Figure 7:**
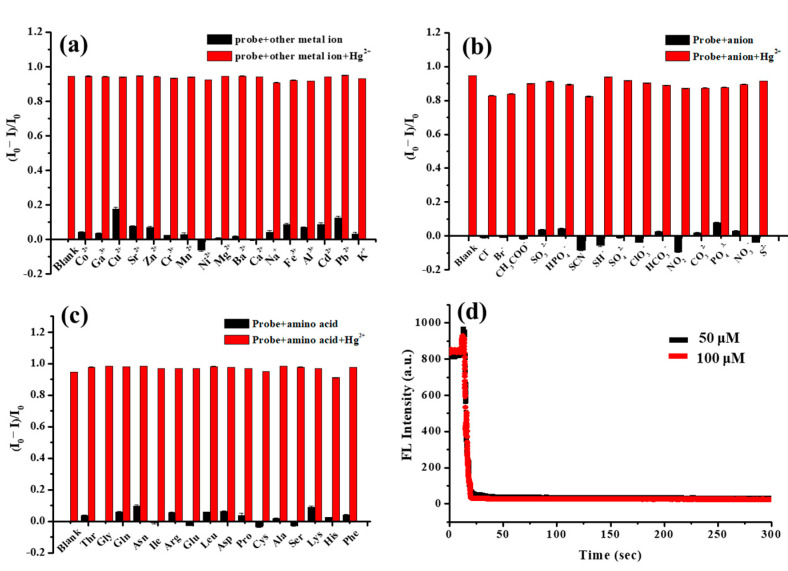
Fluorescence response of FA-PDA FONs probe upon addition of (**a**) 18 metal ions, (**b**) 15 anions, and (**c**) 16 amino acids, respectively, and the concentrations of metal ions, cations, and amino acids are 50 μM. (**d**) FL intensities of FA-PDA FONs probe after addition of 50 μM (black line) and 100 μM (red line) Hg^2+^ as a function of time.

To evaluate the selectivity of FA-PDA FONs based probe, the analyte detecting was assessed. As shown in Figure 7a, most of the metal ions had no or negligible quenching effect except Hg^2+^, and the quenching efficiency of Hg^2+^ could reach 94.38%. The fluorescence quenching maybe due to the electron/energy transfer, because the prepared FA-PDA FONs surface contains such as, oxygen and nitrogen species, and which could preferentially interact with Hg^2+^ [48,49,50]. As shown in Figure 7b,c, 15 anions and 16 amino acids were also used as interfering substances to evaluate the selectivity of FA-PDA FONs. Apparently, the anions and amino acids do not cause tremendous decrease in the fluorescence intensity. By adding Hg^2+^ into every interfering substance test, including metal ions, anions, and amino acids, the fluorescence was significantly quenched, confirming that the prepared FA-PDA FONs probe could selectively detect Hg^2+^ in the presence of interfering substances.

Furthermore, the effect of the response time was also explored to evaluate the sensitivity of the FA-PDA FONs based sensing system as shown in Figure 7d, the change of FL intensity of the FA-PDA FONs probe after the addition of Hg^2+^ was investigated. Clearly, after 10 s of reaction time, the fluorescence intensity dropped to a stable value, while almost unchanged was observed in the next 290 s, which indicates that the FA-PDA FONs could be interacted with Hg^2+^ rapidly, and reached steady states within 10 s.

### 3.4. Fluorescence Sensitivity of FA-PDA FONs toward Hg^2+^

**Figure 8:**
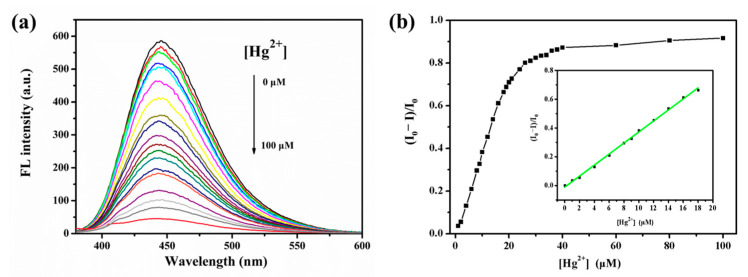
(**a**) Fluorescence emission spectra of the FA-PDA FONs probe with the addition of varying Hg^2+^ concentrations. (**b**) The relationship between the (I_0_ − I)/I_0_ ratio and the Hg^2+^ concentration in the 0–100 μM range. The inset shows the linear relationship between the (I_0_ − I)/I_0_ ratio and the Hg^2+^ concentration in the 0–18 μM range. The regression equation was y = 0.03832x − 0.01093.

To further assess the sensitivity of the prepared FA-PDA FONs probe toward Hg^2+^, the change in the fluorescence intensity of FA-PDA FONs with the concentration of Hg^2+^ were measured. Keeping the concentration of FA-PDA FONs constant, the concentration of Hg^2+^ was increased, and resulted in a step-wise decrease in FL intensity, as shown in Figure 8a. When the added concentration of Hg^2+^ reaches 40 µM, the quenching rate of the probe could reach 94%. Moreover, the Stern–Volmer plots was analyzed, a great linear relationship was obtained between the fluorescence ratios ((I_0_ − I)/I_0_) and Hg^2+^ concentration (0–18 μM) with a correlation efficient (R^2^) of 0.998, as shown in Figure 8b. The limit of detection (LOD) was calculated as 0.18 μM by using the formula LOD = 3σ/k (where σ represents the standard deviation for 20 blank measurements and k represents the slope of the calibration curve) [35]. The FA-PDA FONs based probe towards Hg^2+^ was compared with other reported carbon dots (CDs)-based probes for Hg^2+^ detecting and summarized in Table 1. As can be seen that the FA-PDA FONs based probe has proper and green synthetic method, comparable linear range and relatively low LOD, indicating its possible application to Hg^2+^ sensing in real water samples.

### 3.5. Recovery Tests in Real Water Samples

In order to evaluate the practical applicability of the FA-PDA FONs-based probe, experiments for the selective recognition of Hg^2+^ was performed in mineral water (Hang zhou, China, pH = 6.69 ± 0.13) and tap water (Lu’an, China, pH = 7.35 ± 0.09), respectively. The mineral water and tap water were both filtered by using 0.2 μm syringe filters, and then spiked with a known concentration of Hg^2+^ (5 μM, 10 μM, and 15 μM). Apparently, the percent recovery range for Hg^2+^ is 84.03–97.01%, while the RSDs was less than 3.00% for the Hg^2+^ detection, as shown in Table 2. These results indicated that the proposed method was promising as an extremely selective and sensitive fluorescent probe for the detection of Hg^2+^.

## 4. Conclusions

In summary, we developed a facile and efficient FA-PDA FONs based probe for Hg^2+^ detection, which was prepared by one-pot self-polymerization using FA and DA as precursors. The prepared FA-PDA FONs have a uniform spherical morphology, and the particle size was 1.9 ± 0.3 nm in diameter, which exhibited excellent photostability, high chemical stability and QY. However, the stability of the FA-PDA FONs solution was dependent on pH condition, the precipitation was occurred due to the aggregation of the particles at pH = 2, and the fluorescence intensity was significantly weakened. The FA-PDA FONs can maintain better stability in the pH range of 4–10, which was attributed to the negative charge on the particles surface. In particular, the fluorescence intensity of FA-PDA FONs can be quenched by Hg^2+^ compared to other analytes. Actually, FA-PDA FONs provided a rapid, selective, and sensitive fluorescent probe for detecting Hg^2+^, with an LOD of 0.18 µM. The applicability of the FA-PDA FONs based probe was evaluated with satisfactory recoveries ranging from 84.03–97.01% by detection of Hg^2+^ in spiked real water samples. Furthermore, owing to the facile preparation, good stability, the FA-PDA FONs are expected to have great potential application in chemo/biosensors.

## Figures and Tables

**Table 1:** Comparison of different CDs-based probes towards Hg^2+^.

Precursor	Synthetic Method	Linear Range (μM)	LOD (μM)	References
Folic acid-ethylene glycol	Hydrothermal	0–25	0.23	[51]
Citric acid-urea-cysteine	Microwave	0–40	2.0	[52]
Citric acid-rubeane	Microwave	0–20	0.18	[53]
Citric acid-diethylenetriamine	Reflex	0–80	0.2	[54]
Cysteine	Microwave	1–75	0.5	[55]
Folic acid-dopamine	Self-polymerization	0–18	0.18	This work

**Table 2:** Determination and recovery tests of Hg^2+^ in real water samples (n = 3).

Added (μM)	Mineral Water			Tap Water		
	Found (μM)	Recovery (%)	RSD (%)	Found (μM)	Recovery (%)	RSD (%)
5	4.75 ± 0.11	92.50–96.65	2.15	4.39 ± 0.15	84.47–90.17	3.00
10	8.75 ± 0.12	86.10–88.33	1.23	8.46 ± 0.07	84.03–85.37	0.72
15	14.29 ± 0.31	93.02–97.01	2.04	13.21 ± 0.17	86.92–89.18	1.13

## Data Availability

All data generated or analyzed during this study are included in this article.

## Supplementary Materials

The following supporting information can be downloaded at: https://www.mdpi.com/article/10.3390/polym15081892/s1, Figure S1: The integrated fluorescence (FL) intensity (excited at 350 nm) and absorbance (at 350 nm) of the samples, (A) quinine sulfate; (B–H) from sample #1 to sample #7, respectively. Figure S2: (A) Zeta potential versus pH curves for FA-PDA FONs. The photographs of FA-PDA FONs in different pH solutions under the natural light (B) and the irradiation of 365 nm UV lamp (C), after 3 days of storage. Table S1. The prepared FA-PDA FONs with different mass ratios.
