# Supplementary material for: Folic Acid Adjustive Polydopamine Organic Nanoparticles Based Fluorescent Probe for the Selective Detection of Mercury Ions
Source: Polymers (Basel). 2023 Apr 14;15(8):1892. doi: 10.3390/polym15081892 (PMC10142360; doi:10.3390/polym15081892)
Supplement: Supplementary file 1 [file polymers-15-01892-s001.zip › polymers-2319395-supplementary.pdf]

1. Experimental details

1.1. Synthesis of FA-PDA FONs

The details for synthesis of FA-PDA FONs are listed in Table S1. Folic acid and dopamine·HCl with different mass were dissolved in 100 mL pure water, successively, at room temperature and stirred for 2.5 h, then 0.1504 g Tris was added to above solution to adjust the pH of the reaction solution to 9, and stirred for 5 h in room temperature, after that, the resulting solution was aged for one week. Afterwards, the solution was filtered by 0.22 μm micro-filter membrane and purified in pure water by dialysis through porous cellulose bag (molecular weight cut off 1000 Da) for 4 h. Finally, the products inside the dialysis bag were collected and dried by freeze-drying.

Table S1. The prepared FA-PDA FONs with different mass ratios.

Samples	FA	DA	FA/DA
1#	15 mg	45 mg	1/3
2#	15 mg	30 mg	1/2
3#	15 mg	15 mg	1/1
4#	30 mg	15 mg	2/1
5#	45 mg	15 mg	3/1
6#	60 mg	15 mg	4/1
7#	75 mg	15 mg	5/1

1.2. Quantum yield measurement

The quantum yield (QY) of FA-PDA FONs was calculated by using quinine sulfate (0.1 M H_2SO_4 as a solvent; Φ_{sr} = 54%) as the standard reference material, and the calculation equation is as follows:

$$\Phi_s=\Phi_{sr}\,(k_s/k_{sr})(\eta_s^2/\eta_{sr}^2) \qquad (1)$$

Here Φ is QY, k is the slope, η is the refractive index of the solvent, the subscript sr is the standard reference material, and subscript s is the sample, and for these aqueous solutions, $\eta_s^2/\eta_{sr}^2 = 1$. The slits of excitation and the emission were both set to a width of 5 nm, and the absorbance and fluorescence spectra were recorded under the excitation wavelength at 350 nm. The integrated fluorescence intensities were calculated in the range of 360-600 nm.

2. Figures

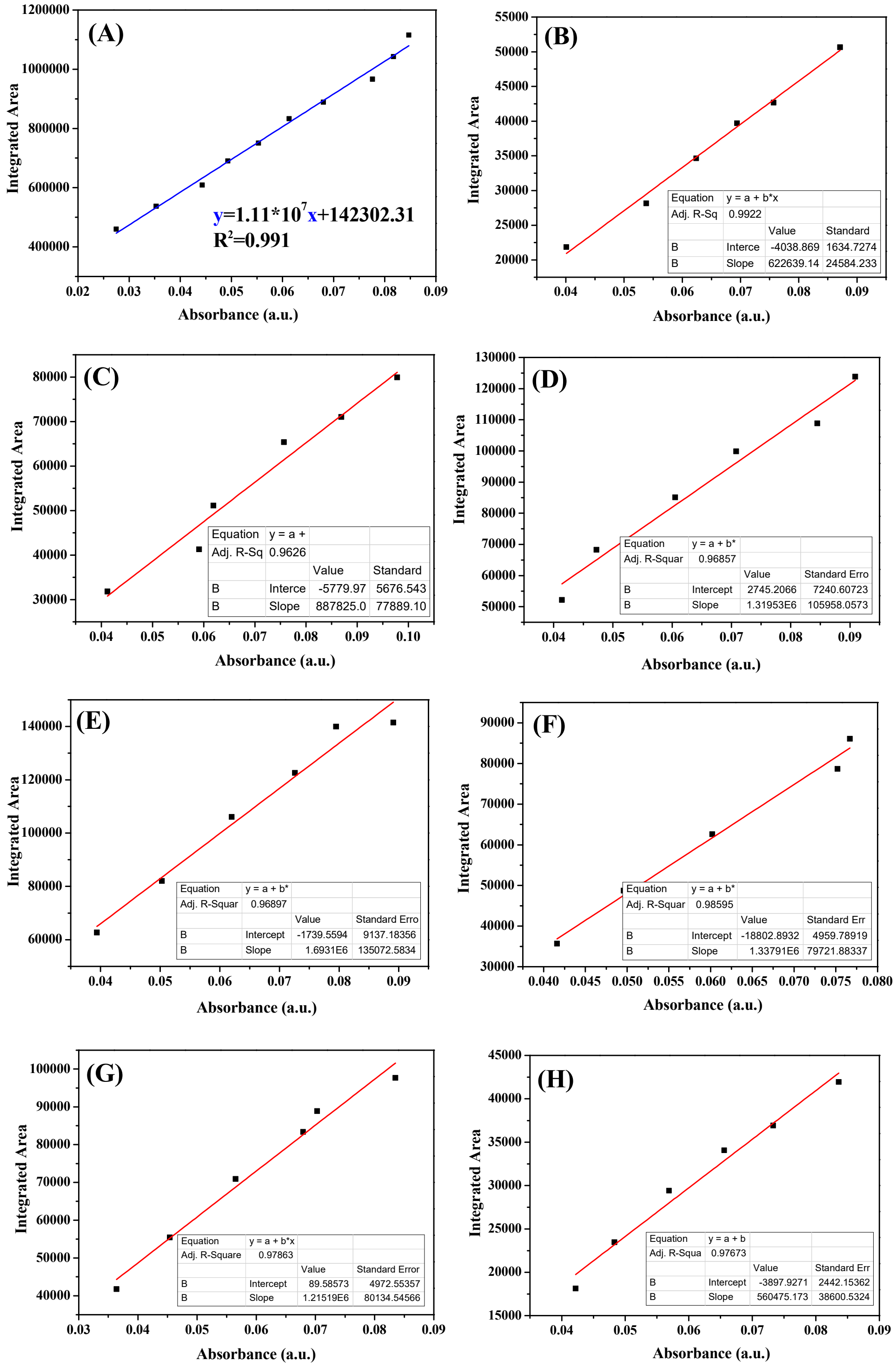

Figure S1. The integrated fluorescence (FL) intensity (excited at 350 nm) and absorbance (at 350 nm) of the samples, (A) quinine sulfate; (B-H) from sample #1 to sample #7, respectively.

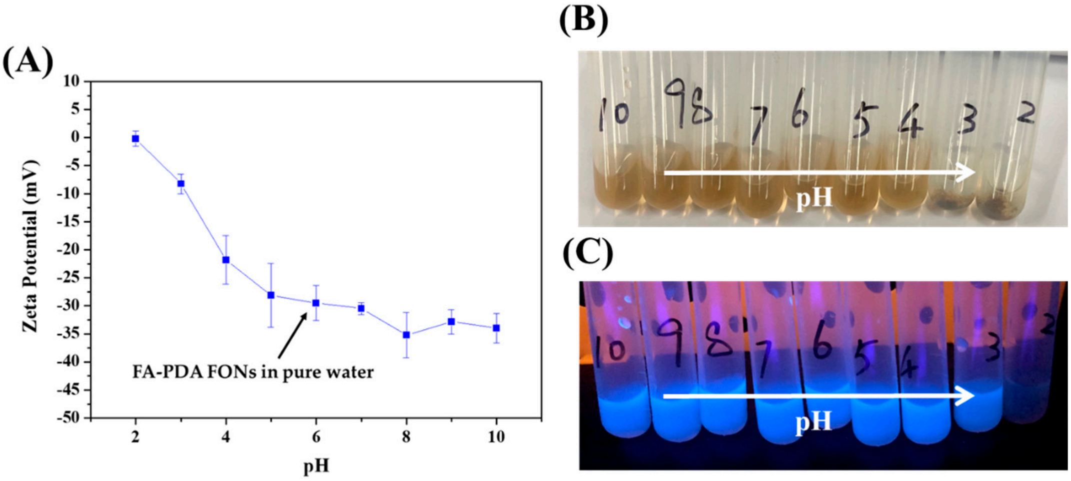

Figure S2. (A) Zeta potential versus pH curves for FA-PDA FONs. The photographs of FA-PDA FONs in different pH solutions under the natural light (B) and the irradiation of 365 nm UV lamp (C), after 3 days of storage.
